# Nuanced HEXACO: A Meta-Analysis of HEXACO Cross-Rater Agreement, Heritability, and Rank-Order Stability

**DOI:** 10.1177/01461672241253637

**Published:** 2024-06-03

**Authors:** Sam Henry, Will Baker, Denis Bratko, Patrick Jern, Christian Kandler, Joshua M. Tybur, Reinout E. de Vries, Laura W. Wesseldijk, Alexandra Zapko-Willmes, Tom Booth, René Mõttus

**Affiliations:** 1 The University of Edinburgh, UK; 2 University of Zagreb, Croatia; 3 Åbo Akademi University, Turku, Finland; 4 University of Bremen, Germany; 5 Vrije Universiteit Amsterdam, The Netherlands; 6 Amsterdam University Medical Center, The Netherlands; 7 Max Planck Institute for Empirical Aesthetics, Frankfurt, Germany; 8 University of Siegen, Germany; 9 University of Tartu, Estonia

**Keywords:** personality structure, HEXACO, Big Five, personality traits, personality nuances

## Abstract

Most Five-Factor Model (FFM) questionnaire items contain unique variance that is partly heritable, stable, and consensually observable, demonstrates consistent associations with age and sex, and predicts life outcomes beyond higher order factors. Extending these findings to the HEXACO model, we meta-analyzed single-item cross-rater agreement, heritability, and 2-year stability using samples from six countries. We analyzed raw item scores and their residual variance and adjusted the estimates for measurement unreliability. The median cross-rater agreement, heritability, and stability estimates were, respectively, .30, .30, and .57, for raw items and .10, .16, and .39, for item residuals. Adjusted for reliability, the respective medians were .46 and .25 for cross-rater agreement, .46 and .39 for heritability, and .87 and .94 for stability. These results are strikingly consistent with FFM-based findings, providing nondismissible evidence that single items index a partly unique level of the trait hierarchy—personality *nuances*—with trait properties comparable to those of higher-order traits.

A key question of personality science continues to be the dimensionality of how people differ in thinking, feeling, behaving and motivation—that is, personality traits. Many scientists have coalesced around the idea that personality variation can be roughly summarized with the Big Few (Mõttus et al., 2020) broad trait domains such as those of the Five-Factor Model (FFM; Costa & McCrae, 1992) or Big Five (Goldberg, 1990) on the one hand or the six-factor HEXACO on the contrary (Ashton & Lee, 2020). These Big Few are sometimes broken into a few dozen aspects or facets, although no consensually agreed model for them exists yet. For example, the Big Five Aspects Scale (DeYoung et al., 2007) contains 10 aspects, the Big Five Inventory (Soto & John, 2017) 15 facets, the NEO Personality Inventory Revised (NEO-PI-R; Costa & McCrae, 1992) 30 facets, the HEXACO Personality Inventory Revised (HEXACO-PI-R; Ashton & Lee, 2007) 25 facets, and the Berlin Multi-Facet Personality Inventory (Rouco et al., 2022) 38 facets. Some have even proposed a 70-facet trait model (Irwing et al., 2023). Regardless of how many and which facets are proposed, these models’ common premise is that facets are pockets of shared variance among some lower-level constituents, typically questionnaire items, that are more specific and numerous than the Big Few domains.

However, growing evidence suggests these lower-level constituents themselves, above and beyond their shared variance, represent specific traits—personality *nuances* (McCrae, 2015)—that capture valid information about individual differences, their causes, development, and consequences (Mõttus et al., 2019). If so, personality traits are best thought of as a truly multilevel hierarchy with five or six very broad domains composed of up to a few dozen narrower aspects or facets, which in turn consist of potentially hundreds of nuances (Condon et al., 2021). So far, however, research on nuances’ trait-like properties has been confined to the FFM (Big Five) assessment frameworks, whereas we aimed to extend these findings to the six-factor HEXACO model. Is HEXACO as nuanced as the FFM?

## Why Care?

The implications of potentially hundreds of nuances being valid personality traits go far beyond psychometric peculiarities. Such a reality would suggest that personality, its underlying causes, and its influence on people’s lives are far higher dimensional than previously thought (Mõttus et al., 2020). This could help explain the field’s modest success in outlining the genetic, neural, and experiential antecedents of personality traits (Avinun et al., 2020; Bühler et al., 2023; Lo et al., 2017). For example, many questionnaire items—markers for nuances—appear to have unique genetic variance components (e.g., Mõttus et al., 2019), associations with brain morphology (e.g., Hyatt et al., 2022), developmental trajectories (Mõttus & Rozgonjuk, 2021), and cross-cultural variations (Achaa-Amankwaa et al., 2021). Likewise, nuances typically help improve personality traits’ predictive validity for life outcomes (Revelle et al., 2021; Saucier et al., 2020; Seeboth & Mõttus, 2018; Stewart et al., 2021) and can explain why traits are linked with them in the first place (Mõttus et al., 2020). Given these early findings related to narrow personality traits, personality research has very good reasons to explore broad trait domains more thoroughly. That the Big Few and their facets provide useful summaries of individual differences *and* that the field can benefit from an increasingly refined understanding of its phenomena are not mutually exclusive goals scientifically, although we realize that many in a field that has only recently coalesced around a few simple models may see more nuance as nuisance. As such, we consider investigating how many and how specific traits the broad personality domains encompass to be a sign of healthy scientific progress that also cautions researchers about the potential of confirmation biases pushing the field toward exclusively low-dimensional trait models.

## What Makes a Unique Trait?

A personality trait should represent a unique aspect of individual differences that is relatively enduring (Funder, 1991), detectable with different methods (Funder et al., 1995; McCrae & Costa, 1987), and a (partly) inherent property of individuals rather than their experiences alone (e.g., Allport, 1931; McCrae & Costa, 2008). These properties, respectively, can be assessed by examining (a) the *rank-order stability* of trait measurements from multiple time points, (b) correlations of individuals’ self-ratings with their ratings by close others (*cross-rater agreement*), and (c) average trait differences between individuals with different levels of familial relatedness (*e.g., heritability*). The traits’ usefulness is also evidenced by their unique developmental trajectories and links with possible antecedents and outcomes (Mõttus et al., 2019).

The Big Few domains are partly stable over time (Terracciano et al., 2006), agreed upon by informants (Connelly & Ones, 2010; De Vries et al., 2008), and heritable (Briley & Tucker-Drob, 2014; De Vries et al., 2022; Kandler et al., 2019;Vukasović & Bratko, 2015). They are also pervasively—albeit generally weakly—correlated with a host of life outcomes (Ozer & Benet-Martínez, 2006; Soto, 2019; Stewart et al., 2021; Zettler et al., 2020), and have cross-culturally replicable associations with demographic factors like sex and age (Allik et al., 2013; Lee & Ashton, 2020). The facets of both HEXACO and FFM tend to have these same properties, even after removing the variance they share with domains (Anglim et al., 2020; Jang et al., 1998; Lee & Ashton, 2018; McCrae et al., 2005), attesting to their trait status.

Several recent studies have found that items in FFM measures display precisely the same empirical properties as the higher order facets and domains they ostensibly index. For example, NEO-PI-R items contain unique variance that is partly heritable, stable over many years, and observable to different raters (Mõttus et al., 2014, 2017), and this replicates across several languages and cultures (Mõttus et al., 2019). Examples of similar studies for HEXACO, on the other hand, are sparse. De Vries et al. (2016) used cross-rater agreement estimates of the HEXACO-PI-R to evaluate a number of item characteristics but did not report the property *per se*. Hang et al. (2021) showed that both HEXACO items and their unique variances predicted age with twice as much accuracy as HEXACO domains, and 39% more accurately than facets—findings much in line with those listed above for FFM items (Mõttus & Rozgonjuk, 2021), and Hofmann et al. (2023) reported similar findings on items’ predictive accuracy for gender in both HEXACO and FFM inventories. However, these are the only existing reports of empirical properties for HEXACO-PI-R items to our knowledge.

Given that HEXACO is one of the most widely used Big Few trait models, we conducted a large-scale cross-cultural meta-analysis (total *N* = 10,958 from Canada, Croatia, Finland, Germany, the Netherlands, and the United Kingdom) on the three key empirical properties of HEXACO-PI-R items: rank-order stability, cross-rater agreement, and heritability. We examined these properties in “raw” item scores as well as their unique variance after having partialed out facets’ and domains’ variance. We also examined items’ meta-analytic associations with sex and age. Finally, using test–retest reliability estimates from previous work (Henry et al., 2022), we dis-attenuated items’ stability, cross-rater agreement, and heritability estimates for random measurement error.

## Material and Method

### Transparency and Openness

We report the origins of our data, all data exclusions, manipulations, and measures in the study. All data, analysis code, and research materials necessary to reproduce the results are available at https://osf.io/kusr5/?view_only=06ba35c8f0444b23b83c79e0d0c9c736. All analyses were conducted in R (R Core Team, 2022), version 4.1.1. This study’s design and its analyses were not preregistered.

For cross-rater agreement and rank-order stability data, we median-replaced missing values of participants with fewer than 10% of missing values in both self- and informant-, twin 1 and twin 2, or time 1 (T1) and time 2 (T2) reports. Participants for whom either source had ≥10% missing values were removed from the sample.^1^ For twin data, we used the same approach when estimating sex and age differences; for estimates of heritability and shared environmental influence, we used full information maximum likelihood estimation.

### Measures

Each sample in the present study used one of three versions of the HEXACO Personality Inventory—Revised (HEXACO-PI-R), which contain 60 (HEXACO-60; Ashton & Lee, 2009), 100 (HEXACO-100; Lee & Ashton, 2018), or 200 (HEXACO-200) items. The HEXACO-200 and HEXACO-100 both assess 25 facets, with eight and four items in each facet scale, respectively. Meanwhile, the HEXACO-60 only assesses the six domains with ten items per scale and thus does not include any items for the interstitial Altruism facet.

### Participants

Lead researchers on the Study of Personality Architecture and Dynamics (SPeADy; Kandler et al., 2019; Wiechers et al., 2023), a study conducted at the University of Bremen, kindly provided us data for heritability, cross-rater agreement, and rank-order stability. SPeADy encompasses two samples, one twin-family sample based on self-reports, and one multirater sample based on self- and informant- reports. Twin data were available for *n* = 1,120 twins, 686 dizygotic (DZ; 188 opposite-sex [OS]) and 498 monozygotic (MZ), for 560 twin pairs. Up to three informant reports were available for *n* = 935 participants (*n* = 1,479 total informants) in a separate (i.e., independent) sample. Of these, *N* = 882 participants also provided self-report data two years later, allowing us to calculate item stabilities, where *n* = 449 of these participants also had informant reports from the first testing instance. All participants completed the HEXACO-60 in German.

Cross-rater agreement data were obtained from samples in Canada, Germany, and the Netherlands. The Canadian sample, originally described in Lee and Ashton (2018), consisted of 2,862 self- and informant reports from an undergraduate student sample who completed the HEXACO-100 in English. The Dutch sample is described in both Allik et al. (2016) and De Vries et al. (2016) and consisted of 2,181 first-year undergraduate students and their informants (friend, family member, or intimate partner) who completed the Dutch HEXACO-200.

Other heritability estimates were calculated using sibling data from Croatia, Finland, and the United Kingdom. In Croatia, 414 twin pairs (total *n* = 828) were recruited to complete the Croatian HEXACO-100, with 147 MZ and 267 DZ (121 OS) pairs. Full details on the Croatian sample can be found in Bratko et al. (2017). Finnish data, originally reported in De Vries et al. (2022), came from a study examining the heritability of personality and political ideology. This sample consisted of 540 MZ and 837 DZ pairs (359 OS), totaling *n* = 1,377 twin pairs who completed the HEXACO-100 in Finnish. Finally, British data (*n* = 3,032 twins) from the “TwinsUK” project (see Lewis & Bates, 2014, for an in-depth description of the sample) consisted of 654 DZ (23 OS) and 873 MZ twin pairs, with all participants completing the English HEXACO-60.

**Figure 1:**
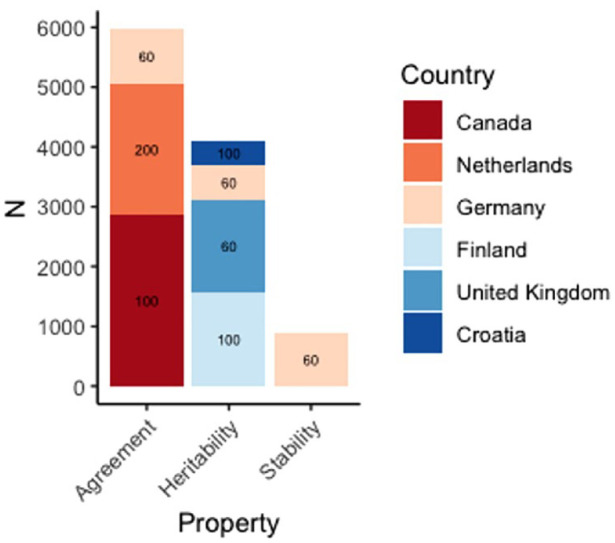
Summary of Samples Used *Note*. Numbers inside each box indicate HEXACO-PI-R version used in each sample

Final samples were thus *N* = 5,978 for items’ cross-rater agreement; *N* = 4,098 informative twin pairs for heritability estimates; and *N* = 882 for 2-year rank-order stability. Comprehensive descriptive statistics for all samples can be found in Table 1 and a graphical summary of these in Figure 1. Power analyses indicated that our samples were all sufficiently large to detect median effects at the magnitudes reported in the meta-analysis of the NEO-PI-*R* (Mõttus et al., 2019).

**Table 1.** Descriptive Information of the Samples.

Variable	Canada	Netherlands	Germany (cross-rater)	Germany (stability)	Croatia	Finland	Germany (twin)	United Kingdom
N	2,862	2,181	935	882	828	3,144	1,140	3,084
Female	1,838	1,794	615	558	523	2,144	828	2,805
Age (*M*)	20.93	20.2	39.44	44.69	22.15	26.41	39.04	57.58
Age (*SD*)	3.91	2.83	18.04	17.46	1.81	7.52	20.19	12.84
Age (range)	14–66	16–56	14–89	14–94	19–28	18–45	14–88	17–85
MZ twin pairs	-	-	-	-	147	590	349	873
Same sex DZ pairs	-	-	-	-	146	557	251	646
Opposite sex DZ pairs	-	-	-	-	121	425	98	23
Number of Items	100	200	60	60	100	100	60	60

*Note.* Cross-rater data are in the first three columns. German cross-rater data included participants with 1, 2, or 3 informant measurements; all others used only one informant. German stability data is a subset of the cross-rater sample.

### Single-Sample and Meta-Analytic Analyses of Agreement, Stability, and Heritability

We calculated cross-rater agreement (*r*_ca_) as the correlation between corresponding self- and informant-report items. We did the same for rank-order stability (*r*_ro_) estimates, pairing self-reports at T1 with self-reports at T2. For twin data, we used ACE variance decomposition techniques with the *umx* package (Bates et al., 2019) to compare correlations of single items between MZ and DZ twins and estimate components of heritability (*h*^2^) as well as shared (*c*^2^) and nonshared environmental (*e*^2^) influences (Mõttus et al., 2017). We used full information maximum likelihood estimation to account for missing data.

All analyses were conducted first on item raw scores, then subsequently on their residual variance after accounting for higher-order variance due to both domains and facets. Specifically, items’ unique variance was obtained by regressing raw item scores on all 25 (or 24, in the case of the HEXACO-60) facets, with the item being residualized omitted from its facet at the time, leaving the leftover variance completely independent of all facets and, consequently, domains.

To calculate meta-analytic estimates for all properties, we used the inverse-variance-based formula (Willer et al., 2010) on the items shared across all datasets for a given criterion. This technique weights estimates by standard errors, giving more importance in final calculations to larger samples. For each item, meta-analytic estimates were calculated based on all available data, meaning that estimates for HEXACO-60 items had larger sample sizes than the additional 40 items for the HEXACO-100.

To calculate statistical significance for meta-analytic estimates, we replicated the approach used by Mõttus et al. (2019) and set a significance level of *p* < .05 after correction for False Discovery Rate (FDR). Mõttus et al. initially used Bonferroni correction in the single samples but switched to FDR after observing a clear pattern of findings across individual samples (i.e., very consistent nonzero estimates for raw items and residuals), and noting that therefore, “using Bonferroni correction in null hypothesis testing may have been too stringent because it assumed that a new *null* hypothesis was tested for each item” (p. e43; emphasis added). That is, given such a high proportion of nonzero estimates in the individual samples, the authors concluded that it was unrealistic for the default null hypothesis of a given estimate of residual cross-rater agreement, rank-order stability, and heritability to be that the estimate was zero. Furthermore, given the wide variety of sample sizes and that our interest was more specifically in the point estimates (and for conciseness), we only report significance for meta-analytic estimates and not individual samples.

### Sex and Age Differences

We then examined how single items’ raw and residual scores related to sex and age. For sex, we calculated Cohen’s *d* by standardizing items’ scores, calculating their means for both sexes, and subtracting the mean of men’s scores from women’s (i.e., positive Cohen’s *d*s can be interpreted as items rated higher by women, where negative effect sizes are those items rated higher by men, on average). For age, we calculated Pearson’s *r*s between age and raw and residual item scores. Positive associations between age and items thus indicate those items that are rated higher by older respondents on average, whereas a negative correlation would indicate the opposite where an item is typically rated higher by younger respondents.

We first conducted these analyses in individual country samples and then meta-analyzed them for the other item properties. This allowed us to consolidate all samples except for the German stability data, which contained participants from the self- and informant-report data. However, due to comparatively limited age ranges in the Dutch, Canadian, and Croatian samples—each with means near 20 years old and *SD*s <5 years, we opted to conduct additional age analyses excluding these samples. Full details comparing single items’ associations with age across different degrees of variation in age—including an additional analysis using only samples with *SD*_Age_ > 10—are contained in the Online Supplement. Here, we only report age associations for samples with *SD*_Age_ > 5 years.

Ultimately, the final analyses of mean sex differences used the full available samples of *N* = 6,851 and *N* = 9,862 for HEXACO-100 and HEXACO-60, while single-item associations with age used samples of *n* = 4,412 for HEXACO-60 and *n* = 1,377 (i.e., the Finnish sample) for HEXACO-100.

### Adjusting Estimates for Error

Estimates of the items’ properties likely contain different sources of error. While many studies have corrected traits’ estimates by dividing them by internal reliability estimates of the traits’ scales, this is impossible for single-item estimates of single-item properties (besides, since internal consistency typically underestimates reliability, this leads to over-corrections). However, data with multiple sources of information, such as short-term test–retest ratings or data from multiple raters, can provide a method of accounting for random and systematic biases to varying degrees (McCrae, 2015; Mõttus et al., 2014, 2017, 2023). Thus, raw and residual meta-analytic estimates for each criterion were first divided by raw and residual 13-day test–retest reliability to dis-attenuate for occasion-specific bias and random error. Then, we estimated the cross-lagged, cross-rater correlations of items, correcting these for concurrent, cross-rater correlations (i.e., *r*_ca_) to dis-attenuate them for both single rater-specific variance and measurement error simultaneously; see Mõttus et al. (2017) for further details on these calculations, and (McCrae, 2015, 2018) for an overview of the variance decomposition model.

## Results

### Cross-Rater Agreement

For raw item scores, estimates of *r*_ca_ ranged from *r*_ca_ = .09 to *r*_ca_ = .57 and had medians of *r*_ca_ = .28, .34, and .31 in Canada, Germany, and the Netherlands, respectively (Table 2). Items’ residual cross-rater correlations ranged from *r*_ca_ = 0 to *r*_ca_ = .44, with a notably higher median in the German twin cohort study sample (*r*_ca_ = .17) than in the Canadian (*r*_ca_ = .08) or Dutch student samples (*r*_ca_ = .11). Items’ raw and residual *r*_ca_s were highly correlated within each sample, with ⍴s = .64, .80, and .77 between the vectors for raw and residual *r*_ca_s of the Canadian, German, and Dutch data, respectively. These generally high associations indicate that the items with comparatively higher consensual validity also demonstrated higher cross-rater agreement once variance shared with higher-order traits was partialed out of their scores. In other words, some items simply were more agreed upon than others regardless of how much they reflect any higher-order trait.

**Table 2.** Raw and Residual Estimates of Cross-Rater Agreement for Three Countries.

	Canada		Germany		Netherlands	
Measure	Raw	Residual	Raw	Residual	Raw	Residual
**Median**	**.28**	**.08**	**.34**	**.17**	**.31**	**.11**
First quartile	.21	.05	.26	.12	.24	.08
Third quartile	.32	.12	.41	.20	.36	.16

### Heritability and Shared Environmental Influences

Tables 3 and 4 contain raw and residual estimates of *h*^2^ and *c*^2^ from Croatian, Finnish, German, and U.K. data. Across all samples, estimates ranged from *h*^2^ = .00 to *h*^2^ = .61 for raw items, with respective medians of *h*^2^ = .32, .29, .33, and .25. Residual estimates ranged from *h*^2^ = .00 to *h*^2^ = .46, with medians of *h*^2^ = .14, .13, .17, and .11 for Croatian, Finnish, German, and U.K. data. Meanwhile, *c*^2^ estimates were generally negligible (Table 4) with median raw and residual c^2^ = .00 in all samples. Raw estimates ranged from *c*^2^ = .00 to *c*^2^ = .31, residuals from *c*^2^ = .00 to *c*^2^ = .23.

**Table 3.** Raw and Residual Estimates of Heritability for Four Countries.

	Croatia		Finland		Germany		United Kingdom	
Measure	Raw	Residual	Raw	Residual	Raw	Residual	Raw	Residual
Median	**.32**	**.14**	**.29**	**.13**	**.33**	**.17**	**.25**	**.11**
First quartile	.24	.06	.23	.08	.22	.08	.20	.07
Third quartile	.41	.23	.36	.17	.40	.23	.29	.16

**Table 4.** Raw and Residual Estimates of Shared Environmental Influence for Four Countries.

	Croatia		Finland		Germany		United Kingdom	
Measure	Raw	Residual	Raw	Residual	Raw	Residual	Raw	Residual
Median	**0**	**0**	**0**	**0**	**0**	**0**	**0**	**0**
First quartile	0	0	0	0	0	0	0	0
Third quartile	0	.05	0	.03	.02	.05	0	.02

Correlations between raw and residual *h*^2^ estimates were consistent with those for cross-rater agreement: ⍴s = .63, .52, .74, and .68 in the samples from Croatia, Finland, Germany, and the United Kingdom. This pattern was more moderate for shared environmental influence estimates, with the vectors of raw and residual scores for the respective shared environmental estimates correlating ⍴ = .34, .19, .33, and .52. This suggests that although the estimates were small for the role of the shared environment in personality traits, there was something replicable about these modest estimates.

### Rank-Order Stability

Self-report-based rank-order stability had medians of *r*_ro_ = .57 (range = .38-.78; *IQR* = .51-.62) and *r*_ro_ = .39 (range = .23-.69; *IQR* = .33 to .47) for raw item scores and residuals. Raw and residual estimates correlated ⍴ = .81, indicating that stability, too, appears to be largely a property of the item itself rather than its ability to index higher-order facets and domains.

### Meta-Analysis of Raw and Residual Agreement, Heritability, and Influence of Shared Environment

Before conducting the meta-analysis, we examined correlations between vectors of *r*_ca_ and *h*^2^ across samples to ensure sufficient cross-country consistency for each property (e.g., correlating items’ *r*_ca_ estimates from Canada with those from the Netherlands). Magnitudes of these correlations ranged from moderate to high across samples of both *r*_ca_ and *h*^2^ for raw and residual scores in the HEXACO-60 and HEXACO-100. The correlations between *h*^2^ estimates for HEXACO-100 items from the Croatian and Finnish samples were ⍴s = .38 and .28 (*k* = 100) for raw items and residuals, indicating modest consistency of items’ heritability across the two samples. The *h*^2^ estimates for only HEXACO-60 items showed greater consistency across samples, with the British, Croatian, Finnish, and German samples correlating from ⍴ = .37 to ⍴ = .58 for raw items, but only ⍴ = .05 to ⍴ = .52 for residuals. The cross-sample correlations were higher still for *r*_ca_, with ⍴ = .82 between raw and ⍴ = .68 between residual item scores for the Canadian and Dutch samples. Adding in the German sample that used the HEXACO-60, the final range of associations was ⍴ = .73 to ⍴ = .82 for raw items, and from ⍴ = .53 to ⍴ = .68 for residuals. Given these intercorrelations ranged from medium to large, and that the general distribution of estimates was consistent across samples, we found it justifiable to meta-analyze the findings.

Table 5 contains a summary of meta-analytic estimates of *r*_ca_, *h*^2^, and *c*^2^, as well as the single-sample *r*_ro_, for raw and residual scores of HEXACO-PI-R items. Detailed findings of all estimates reported in this section can be found in the Online Supplement. The appendix reports raw and residual meta-analytic estimates of *r*_ca_, *r*_ro_, *h*^2^, and *c*^2^ as well as the standard deviation and short-term test–retest reliability for all HEXACO-100 items.

**Table 5.** Meta-Analytic Estimates for Cross-Rater Agreement, Rank-Order Stability, Heritability, and Shared Environmental Influences for HEXACO-PI-R.

	Raw item scores				Residual item scores			
Measure	*r*_ca_	*h* ^2^	*c* ^2^	*r*_ro_^a^	*r*_ca_	*h* ^2^	*c* ^2^	*r*_ro_^a^
Median	.30	.30	0	.57	.10	.16	.02	.39
First quartile	.22	.25	0	.51	.07	.13	0	.33
Third quartile	.35	.36	.03	.62	.14	.21	.07	.47
Proportion significant	100%	98%	0%	100%	98%	69%	3%	100%

*Note. r*_ca_ = cross-rater agreement. *h*^2^ = heritability. *c*^2^ = influence of shared environment. *r*_ro_ = 2-year rank-order stability. Proportion significant = the percentage of estimates significant at *p* < .05 after FDR correction for multiple testing.

a Stability estimates are (a) not meta-analytic and (b) only summarize findings for the HEXACO-60.

For the *r*_ca_ and *h*^2^ of raw items, both had meta-analytic medians = .30, whereas item residuals had median *r*_ca_ = .10 and median *h*^2^ = .16. Raw agreement ranged from *r*_ca_ = .10 to *r*_ca_ = .48, residuals from *r*_ca_ = .02 to *r*_ca_ = .30. Meanwhile, *h*^2^ for raw items ranged from *h*^2^ = .17 to .50; residuals ranged from *h*^2^ = .01 to *h*^2^ = .40.

Among *r*_ca_ estimates, 100% of raw and 98% of residual estimates were significant. For raw item scores, 98% demonstrated significant *h*^2^, while 69% of residuals were significantly heritable after FDR correction. As in individual samples, the meta-analytic shared environmental influence estimates were mostly negligible, with medians of *c*^2^ = .00 and *c*^2^ = .02 for raw and residual estimates. These estimates were also similar in their distributions: raw estimates ranged from *c*^2^ = .00 to *c*^2^ =.19; estimates for residuals ranged from *c*^2^ = .00 to *c*^2^ = .19. Just three-item residuals—but no raw items—reached FDR-corrected significance for the influence of shared environment.

The results of our meta-analysis are strikingly similar to those for the NEO-PI-R as reported in Mõttus et al. (2019); these estimates are reported in Table 6. They found median raw *r*_ca_ = .28 (*IQR* = .23 to .33), *h*^2^ = .28 (*IQR* = .23 to .33), and *c*^2^ = .00 (*IQR* = 0 to 0); and median residual *r*_ca_ = .12 (*IQR* = .09 to .16), *h*^2^ = .14 (*IQR* = .07 to .18), and *c*^2^ = .00 (*IQR* = 0 to 0). The stability estimates reported for the NEO-PI-R were slightly lower than those for the HEXACO-PI-R, with median raw *r*_ro_ = .41 (*IQR* = .34 to .45) and residual *r*_ro_ = .24 (*IQR* = .20 to .29). This is most likely due to the shorter interval between measurements, while Mõttus et al. used measurements taken up to 16 years apart, estimates here are based on a single 2-year interval.

**Table 6.** Meta-Analytic Estimates, Taken From Mõttus et al. (2019) for Cross-Rater Agreement, rank-Order Stability, Heritability, and Shared Environmental Influences for the NEO-PI-R.

	Raw item scores				Residual item scores			
Measure	*r*_ca_	*h* ^2^	*c* ^2^	*r*_ro_	*r*_ca_	*h* ^2^	*c* ^2^	*r*_ro_
Median	.28	.28	0	.41	.12	.14	0	.24
First quartile	.23	.23	0	.34	.09	.07	0	.20
Third quartile	.33	.33	0	.45	.16	.18	0	.29
Proportion significant	100%	98%	0%	100%	97%	70%	3%	100%

*Note. r*_ca_ = cross-rater agreement. *h*^2^ = heritability. *c*^2^ = influence of shared environment. *r*_ro_ = rank-order stability up to 16 years. Proportion significant = the percentage of estimates significant at *p* < .05 after FDR correction for multiple testing.

### Associations Between Item Properties

We also examined how items’ empirical properties were associated with one another. Table 7 contains these correlations between vectors of meta-analytic estimates for HEXACO-100 item properties juxtaposed with the same estimates reported for the NEO-PI-*R* (Mõttus et al., 2019). For HEXACO-100 items, we also included estimates of items’ test–retest reliability (*r*_tt_) and standard deviation (*SD*), two properties that have previously been shown to track strongly with validity criteria (Mõttus et al., 2019).

**Table 7.** Correlations Between the Item-Level Estimates for HEXACO-PI-R and NEO-PI-R Cross-Rater Agreement, Rank-Order Stability, Heritability, and Shared Environmental Influences

	HEXACO-PI-R								NEO-PI-R		
Measure	*r*_ca_	*r*_ro_^a^	*h* ^2^	*c* ^2^	*SD*	*r*_tt_		*r*_ca_	*r*_ro_	*h* ^2^	*c* ^2^
*r*_ca_	**.80**	.73	.61	−.01	.66	.62	*r*_ca_	**.58**	.36	.39	−.11
*r*_ro_^a^	.68	**.81**	.50	0	.58	.56	*r*_ro_	.43	**.68**	.47	−.07
*h* ^2^	.65	.36	**.68**	−.11	.50	.56	*h* ^2^	.42	.46	**.73**	−.45
*c* ^2^	−.06	−.11	−.29	**.68**	−.10	0	*c* ^2^	−.06	−.12	−.52	**.59**
*SD*	.48	.35	.45	−.11	**.83**	.49					
*r*_tt_	.63	.57	.48	−.12	.45	**.70**					

*Note. r*_ca_ = cross-rater agreement. *r*_ro_ = rank-order stability. *h*^2^ = heritability. *c*^2^ = shared environmental influence. *SD* = standard deviations. *r*_tt_ = test–retest reliability. Correlations for estimates of item residuals are below the diagonal; correlations for estimates of raw items’ scores are above the diagonal. On the diagonals are the correlations between respective estimates from items’ raw and residual scores.

a Correlations with HEXACO-PI-R rank-order stability are for the HEXACO-60 only.

We calculated meta-analytic *SD*s using the seven independent samples in the present study (i.e., excluding German stability data). In individual samples, we estimated the *SD*s of items’ raw scores as the mean of both data sources/assessment occasions in each sample (i.e., Twin 1 and Twin 2, self and informant, and Time 1 and Time 2). Residual *SD*s were calculated for the items’ unique variance. We then took a weighted average of each item’s raw and residual *SD* to use for the present calculations. Median single item *SD* for HEXACO-100 items were *SD* = .82 (*M* = .83, *SD* = .11, range = .50–1.08) for raw items and *SD* = .63 (*M* = .63, *SD* = .08, range = .42–.84) for their residual variance.

For *r*_tt_, raw estimates were taken from Henry et al. (2022), who reported on the short-term (~13-day) retest reliability of the HEXACO-100 items (*N* = 416 recruited from Prolific Academic); estimates of items’ residual *r*_tt_s were calculated using raw data from the Supplemental Materials of Henry et al., available at https://osf.io/wz3du/?view_only=4a2aea689e6b434c84406874eabcfd8f. Median single item *r*_tt_ for HEXACO-100 items were *r*_tt_ = .66 (*M* = .65, *SD* = .08, range = .39–.84) for raw items and *r*_tt_ = .43 (*M* = .43, *SD* = .11, range = .15–.71) for their residual variance.

Overall, HEXACO-100 items’ *h*^2^, *r*_ca_, and *r*_ro_ had substantial intercorrelations, ranging from ⍴s = .50 to .74 for raw items’ estimates and from ⍴s = .36 to .68 for item residuals. Meanwhile, *c*^2^ had near-zero and often *negative* associations with all other properties, reaching as low as ⍴ = −.29 between residual *h*^2^ and residual *c*^2^ in the HEXACO-100. As in the individual samples, associations between raw and residual meta-analytic estimates are highly correlated, ranging from ⍴ = .58 to ⍴ = .81. This shows that items tend to retain their properties even if what they were originally intended to measure—the HEXACO domains and facets—is stripped away.

These associations are largely consistent with those for the NEO-PI-*R* (Mõttus et al., 2019), who found correlations between items’ raw and residual scores for the same property ranged from ⍴ = .58 (*r*_ca_) to ⍴ = .73 (*h*^2^). Associations between properties were generally a bit more modest in the NEO-PI-R for raw item scores: All correlations were ⍴ < .50, whereas those observed for the HEXACO-PI-R had a minimum ⍴ = .53. NEO-PI-R associations for estimates from residual scores more closely resemble ours, ranging from ⍴ = .42 to ⍴ = .46 between *r*_ca_, *r*_ro_, and *h*^2^. Meanwhile, *c*^2^ shows near-zero or negative associations with the other properties in both NEO-PI-R and HEXACO-PI-R. This was especially true for correlations with *h*^2^ in the former, where the two properties correlated ⍴ = −.45 and ⍴ = −.52 for raw and residual scores.

Items’ *SD*s and *r*_tt_s both tracked strongly with the other properties as well, ranging from ⍴ = .50 to ⍴ = .66 for raw items and ⍴ = .35 to ⍴ = .63 for their residual variance—very similar in magnitude to the positive manifold observed among *h*^2^, *r*_ca_, and *r*_ro_. Both properties, and especially *SD*, also had very high correlations between their raw and residual variance (⍴ = .83 for *SD* and ⍴ = .70 for *r*_tt_) meaning that items with more overall variance to start with also tended to have the most variance beyond higher-order trait variance; similarly, the most stable raw items tend to be those whose unique information is also stable over short periods of time. *SD* and *r*_tt_ were also moderately correlated with each other, with ⍴ = .49 for raw items and ⍴ = .45 for residuals.

Given the high correlations between these properties, we conducted two principal component analyses on the raw and residual correlations from Table 7 (excluding *c*^2^); a one-component solution explained 67% of the variance in correlations between raw properties and 61% for residual properties. We then calculated a composite “informativeness” score for each item, using the mean of their PC scores on standardized estimates for each property, to get an idea of what kinds of content relates to higher/lower levels of desirable empirical properties. For example, the least informative HEXACO-100 item by this metric was “I wouldn’t want people to treat me as though I were superior to them,” while the most informative item was “If I had the opportunity, I would like to attend a classical music concert.” Interestingly, four of the five most informative items were from the Openness to Experience factor.

## Associations With Sex and Age

Across all seven datasets, sex differences between raw item scores ranged from Cohen’s *d*s = -.86 to .96, with residuals ranging from *d* = −.38 to *d* = .37. Sex differences tended to track across samples as well, with median ⍴ = .77 (*M* = .76, range = .56–.93) for raw item scores and median ⍴ = .40 (*M* = .40, range = .14 to .63) for residuals. Meta-analytic sex differences for raw items ranged from *d* = –.58 to *d* = .86, with an absolute median of *d* = .19 (*M* = .25, *IQR* = .09-.36). Item residuals ranged in their differences between men and women from *d* = –.23 to *d* = .27; median absolute differences were small,|*d|* = .05 (*M* = .06, *IQR* = .02-.09). After correcting for False Discovery Rate for the 200 associations estimated across the two demographic variables, 91 and 71 of the 100 estimated sex differences for raw and residual estimates, respectively, were significant. As in the empirical properties above, sex differences in items’ raw and residual scores were correlated (⍴ = .67). In other words, while the magnitude of the sex differences was attenuated from raw to residual items, there appeared to be a substantial amount of discerning information at the level of the residual between men and women.

Associations ranged from *r* = −.19 to *r* = .20 and *r* = −.13 and *r* = .13 for raw and residual item scores across the four samples with *SD*_age_ > 5 years, with 70 raw and 50 residual significant associations after FDR corrections for the 200 associations. The absolute median -associations of age and items for the HEXACO-100 were *r* = .06 (*M* = .07, *IQR* = .04-.10) for raw scores and *r* = .04 (*M* = .04, *IQR* = .02-.07) for residuals. Across the four datasets, correlations between raw item scores and age had median ⍴ *=* .52 (*M* = .54, range = .20-.90), while the same inter-sample correlations for residuals had median ⍴ = .45 (*M* = .46, range = .16-.84). Age differences between items’ raw and residual scores were even more highly correlated than those of sex (⍴ = .84), suggesting that much of the age-relevant information in single items is due to the unique trait they assess.

The five items with residuals most strongly associated with sex and age, respectively, are presented in Table 8 alongside estimates of these differences in raw item scores; a full table of these differences is provided in the Online Supplement. In line with the high correlations between raw and residual scores, all differences were in the same direction. With only a few exceptions, though, items’ residual variance demonstrated a similar—and sometimes stronger—age or sex difference than their raw scores did. This adds further weight to the suggestion that most information on demographic differences is carried by the nuanced trait information indexed by items themselves rather than their higher order traits.

**Table 8:** Items With Residuals Most Strongly Associated With Sex and Age

Sex				
	Raw		Residual	
	*d*	*SE*	*d*	*SE*
*I feel like crying when I see other people crying*	.82	.01	.27	.01
*I would be very bored by a book about the history of science and technology*	.58	.01	.23	.01
*I would be quite bored by a visit to an art gallery*	−.29	.01	−.23	.01
*I would feel afraid if I had to travel in bad weather conditions*	.66	.01	.22	.01
*People often joke with me about the messiness of my room or desk*	−.01	.01	−.16	.01
Age				
	Raw		Residual	
	*r*	*SE*	*r*	*SE*
*I would be tempted to buy stolen property if I were financially tight*	−.06	.03	−.13	.03
*I would like to live in a very expensive, high-class neighborhood*	.18	.03	.13	.03
*I think of myself as a somewhat eccentric person*	−.19	.03	−.13	.03
*I often check my work over repeatedly to find any mistakes*	−.17	.03	−.11	.03
*I get very anxious when waiting to hear about an important decision*	−.19	.03	−.11	.03

*Note. d* = Cohen’s *d* difference between men and women; positive values indicate items where women score higher and men lower, and vice versa. *r* = correlation between age and mean item score; positive correlations indicate higher scores for older individuals and lower scores for younger individuals, and vice versa. *SE* = standard error. All *ps* < .001.

For both sex and age, the pattern of associations with items’ residuals was similar to those found by Mõttus et al. (2019) for the NEO-PI-R. In both studies, the most extreme residual associations with age and sex were around|*r*| = .10 and|*d*| = .20, respectively. Although the NEO-PI-R contains 140 more items than the most-commonly used HEXACO-PI-R inventory, we found a very similar proportion of significant associations, with 47% of residuals significantly associated with age in the analysis using datasets with *SD*_age_ > 5 years (compared with 41% of residuals when using all samples) compared with 43% in the NEO-PI-R. We found proportionally more significant differences between item residuals and sex (71% in the present study vs. 44% in the original meta-analysis), although our analyses here had a greater statistical power to detect significant associations, with *N*s up to 9,862 compared with *N* = 6,287 in the meta-analysis of NEO-PI-R items. Interestingly, when converting between Cohen’s *d* and the Pearson correlation coefficient, the median estimates, as well as the distributions, of sex and age associations with both raw items and residuals are nearly identical in magnitude. Single items thus appear to consistently capture unique, albeit small, differences in meaningful demographic variables.

## Adjusting HEXACO-PI-R Estimates for Unreliability

As the estimates of agreement, stability, heritability, and shared environmental influence were all attenuated by random error—especially their residual variance, which contained all the random error—we approximated more accurate estimates by dividing the observed estimates of empirical properties by their short-term test–retest reliability (*r*_tt_). Presumably, no item property can exceed the item’s reliability. Mõttus et al. (2019) conducted a similar adjustment, although they used *r*_tt_ estimates taken from a different FFM measure and only adjusted the median values for raw and residual estimates of empirical properties. Here, we report only the median-adjusted values for HEXACO-100 items; detailed (i.e., item-level) adjusted results are available in the Online Supplement.

For HEXACO-100 items, medians of adjusted meta-analytic estimates of raw items were *r*_ca_ = .46, *r*_ro_ = .87, *h*^2^ = 0.47, and *c*^2^ = 0, while reliability-adjusted estimates for residuals were *r*_ca_ = .25, *r*_ro_ = .94, *h*^2^ = 0.40, and *c*^2^ = 0.02. The raw estimates are very similar to those of the NEO-PI-*R* (Mõttus et al., 2019) for agreement and heritability (*r*_ca_ = .42, *h*^2^ = 0.42), while the stability estimates were unsurprisingly lower for the NEO-PI-R given a longer retest interval (12-year adjusted *r*_ro_ = .62). Meanwhile, NEO-PI-R items’ residual variance had cross-rater agreement very similar to that observed for HEXACO-100 items (*r*_ca_ = .24) but lower heritability (*h*^2^ = .28) and stability (*r*_ro_ = .48). While the lower stability is to be expected, the disparity in residual heritability estimates may require further investigation. Mõttus et al. note that their estimate of residual *r*_tt_ = .50 was likely an overestimate because the items were residualized only for the FFM domains (facets were not available for the questionnaire) and could therefore “have been inflated by facet-level variance” (p. e46, Mõttus et al., 2019)—our estimated residual *r*_tt_ = .43 supports this claim. As such, they likely under-adjusted due to an unduly high divisor. Finally, Mõttus et al. did not adjust raw or residual estimates of shared environmental influence, as both had medians of *c*^2^ = 0.

## Adjusting for Cross-Method Variance

Adjusting for random measurement error using *r*_tt_ only accounts for occasion-specific and random measurement error but not any stable method effects of each item: *r*_tt_ represents an individual’s biased view of themselves that is stable over time. Having both self- and informant reports at two time points, however, allowed us to examine rank-order stability without the influence of random measurement error and systematic biases associated with a single source (e.g., acquiescent or socially desirable responding). Thus, to account for the possible inflationary effects of method variance on stability estimates, we divided (*cross-time*, cross-rater) correlations (i.e., correlations between self-ratings at T2 and informant-ratings at T1, which are free of stable single-rater influences but deflated by imperfect cross-rater agreement, random error and true trait change) by (*same-time*, cross-rater) correlations—the latter of which were only deflated by random error and imperfect cross-rater agreement but *not* by true change (which we might expect across a 2-year measurement interval). This procedure allowed us to estimate the true rank-order stability of HEXACO-PI-R items free of method effects and measurement error,^2^ a replication of analyses conducted by Mõttus et al. (2017).

Specifically, we used a subsample from SPeADy (*n* = 449) that contained both informant reports on one occasion and self-reports at both T1 and T2 to estimate items “true” rank-order stability. For raw item scores, the concurrent *r*_ca_ estimates were nearly identical to the cross-lagged ones: while concurrent scores had median *r*_ca_ = .35 (range = .12-.59, *IQR =* .25-.42), the cross-lagged estimates had median *r*_ca_ = .33 (range = .08 to .57, *IQR* = .27-.40), and the two vectors correlated ⍴ = .92. This alone speaks to the overall consistency of consensually valid variance of self-reports across the measurement interval; as shown before, the reliable variance in self-reports was also largely stable over time. When we corrected cross-lagged correlations of raw item scores for method effects (i.e., their concurrent cross-rater correlations), the resultant estimates were similar to corrections for retest reliability. Median corrected cross-rater estimates for raw items were $\hat{r}$ _ro_ = .97 (range = .68 to > 1, *IQR* = .85 to > 1); 22 items had $\hat{r}$ _ro_ >1 (expectedly, if true stability was 1.0, then its estimated values would vary around 1.0).

For item residuals, concurrent *r*_ca_ estimates were again quite similar to cross-lagged *r*_ca_, where the latter had median *r*_ca_ = .15 (range = −.04 to .46, *IQR* = .11 to .20) and the former had median *r*_ca_ = .17 (range = .05- 44, *IQR* = .11-.22). After dividing residuals’ cross-lagged correlations by their respective estimates of cross-rater agreement, residuals had median cross-lagged corrected estimates of $\hat{r}$ _ro_ = .83 (range = -.66 to > 1, *IQR* = .67 to > 1); 18 items had corrected cross-lagged $\hat{r}$ _ro_s > 1. Items that had inflated scores tended to be the same for both raw and residual estimates, with the two vectors correlating ρ = .58, and items with the most extreme values (with corrected, cross-lagged $\hat{r}$ _ro_s > 1) were the same in both raw and residual estimates.

The medians of these results are consistent with those correcting stability estimates for reliability: reliable and consensually valid variance in personality test items and even in their unique variance—after higher-order trait variance has been removed from them—is remarkably stable over 2 years, with average correlations nearly .90 or even higher. We warn that the individual estimates resulting from such corrections are likely noisy due to sampling error in both longer-term stability estimates and reliability estimates; however, the median-across-items corrected estimates should be reliable.

## Discussion and Conclusion

Many personality scientists have long assumed that the majority of personality trait variance can be captured by a small number of higher-order factors and perhaps a few dozen of their facets. However, numerous recent FFM-based studies have shown that there is more to personality traits: individual questionnaire items—even if not designed to measure specific traits—often contain unique variance with properties expected of traits. Individual differences in the FFM items’ unique variance are moderately stable over many years, thus not reflecting transient error, and at least partly agreed upon by different raters, thus representing more than idiosyncratic trait perceptions (Mõttus et al., 2019). Moreover, biological relatives tend to be more similar than strangers in the items’ unique variance, suggesting the items capture partly unique etiology and thus, again, represent more than idiosyncratic trait perceptions (Mõttus et al., 2019). Items’ unique variance often also predicts life outcomes, sometimes more so than the traits for which the items were written (Stewart et al., 2021), besides unique developmental trends (Hang et al., 2021) and variations across cultures (Achaa-Amankwaa et al., 2021).

So far, this evidence has been restricted to the FFM trait model, but we here demonstrate that the items designed to measure the HEXACO domains and facets also have unique trait properties similar to the Big Five items. We think that this is how rigorous empirical science should proceed: Carving out a phenomenon—here, personality traits forming a hierarchy that extends below two or three dozen of facets—and using multiple measurement approaches, datasets, cultures, and languages to empirically explore it. Strikingly, when accounting for measurement error, the average HEXACO item and its unique variance were moderately agreed upon by different raters, had a heritability estimate similar to broad personality domains (Vukasović & Bratko, 2015), and were highly stable over time, hence closely replicating the previous FFM-based findings.

At this point, it is no longer clear what further *empirical* evidence would be necessary to accept that unique personality traits exist beyond a few broad constructs each composed of a handful of facets. On empirical grounds, it seems impossible to dismiss items’ unique variance as error or nuisance variance, because otherwise broader higher-order constructs should be dismissed on the same grounds. We are very careful to point out, however, that our findings do not negate the value of the broad constructs typically used to operationalize personality. Instead, they underscore the importance of treating personality traits as truly hierarchical constructs with many equally valid levels of abstraction. Choosing which trait hierarchy level—domains, facets or nuances—to focus on in any given empirical study is a choice researchers explicitly have to make based on their goals (Mõttus et al., 2020). Implicitly assuming that, say, the Big Few are empirically somehow more trait-like than lower hierarchy levels no longer seems justifiable.

### Striking Similarity to the NEO-PI-R

We tested whether HEXACO items demonstrate similar empirical properties to those of one of the most popular and comprehensive FFM questionnaires, the NEO-PI-R. We observed remarkable similarity, with average cross-rater agreement, heritability, and the effect of shared-by-twin-siblings environment falling within just a few correlation or percentage units from previous findings. Only rank-order stability differed noticeably, likely because the measurement interval used in Mõttus et al. (2019) was six times longer on average than the sample used in the present study. Despite the NEO-PI-R using 240 items (compared to the 60- or 100-item HEXACO questionnaires predominantly utilized in the present study), even the proportion of significant findings differed by fewer than two percentage points in most cases, with some exceptions for the shared environment. We are thus confident that HEXACO-PI-R items, as well as their unique variance, demonstrate empirical trait properties very similar to the popular FFM questionnaire, the NEO-PI-R. Is HEXACO as nuanced as the FFM? Our evidence certainly suggests it is.

However, some HEXACO proponents claim that the HEXACO domains capture more personality variance than the FFM, thus better encompassing the personality trait space. For example, Lee et al. (2022) and Thielmann et al. (2021) showed that HEXACO traits predict most of the variance of those in FFM, but not vice-versa. However, greater *higher-*order coverage of the personality space need not necessarily imply any difference in the amount of net information captured at the lowest level of measurement. Just as HEXACO domains may capture more variance than FFM domains, HEXACO items may index more, less, or a comparable amount of unique personality information as FFM items. As it turns out, HEXACO-PI-R item residuals demonstrate almost *exactly* the same heritability, cross-rater agreement, stability, and even associations with sex and age as the NEO-PI-R.

Our findings are thus consistent (or at least not inconsistent) with the HEXACO domains providing an as-good and possibly even better model for parsimoniously describing individual differences than those of the FFM. They do, however, suggest that as one goes further and further down the trait hierarchy, personality is structured in far more complex ways so that differentiating between five or six broad factors at the top does not make much meaningful difference in the sheer amount of information that a test captures in a person (e.g., see Hang et al., 2021 regarding the capture of age differences). In other words, *items capture traits, regardless of the instrument they belong to.* So, besides pitting different Big Few models against each other, researchers could seek to expand our understanding of how much meaningful personality information tests *can* capture in individual differences. One starting point might be exploring how and why individual items vary in their ability to index unique personality information.

### High Empirical Overlap Among Empirical Properties r_ca_, r_ro_, h^2^, *SD*, and r_tt_

The importance of understanding what causes items to be more informative is particularly relevant given the positive manifold observed among items’ desirable empirical properties (Table 7). Across both the HEXACO-PI-R and NEO-PI-R, items that demonstrate high levels of any given empirical property (a) tended to display high levels of others and (b) continued demonstrating that property even with higher-order trait variance removed. This is a consistent finding, robust across inventories and samples (e.g., De Vries et al., 2016; Henry et al., 2022; Henry & Mõttus, 2020; Mõttus et al., 2019). All else held equal, then, some items may simply be more informative about stable and consensually valid individual differences than others, and it appears that this general informativeness is at least partly a property of the unique trait information that the individual items index—not just a reflection of the properties of the higher-order traits the items were initially written to assess. Instead of exclusively focusing on broader traits, we, therefore, suggest that questionnaire constructors who want to optimally capture valid individual differences explicitly prioritize items (and thereby nuances) that are individually as informative as possible—showing high retest reliability, longer-term stability, cross-rater agreement, and familial similarity, among other things.

Previous evidence suggests that items’ variance tracks with other empirical properties such as retest reliability and cross-rater agreement (De Vries et al., 2016; Henry et al., 2022; Mõttus et al., 2019). This finding was both replicated and extended here: item *SD*s were a consistent predictor of all validity criteria (for raw scores and residuals) as well as their reliability (*r*_tt_). In other words, items eliciting more variable responses (i.e., high *SD*s) tend to capture more unique signal about individuals. While the present findings cannot tease apart which property causes another, variance (and short-term stability, to some extent) is arguably the easiest target for investigation. As one example, De Vries et al. (2016) predicted items’ *SDs* using characteristics such as length, negation, evaluativeness, position in survey, and observability, but explained relatively little variance (*R*^2^ = .17 for the NEO-PI-R and *R*^2^ = .06 for the HEXACO-PI-R).

So what causes items to vary in the first place? De Vries et al. (2016) suggested that items with high variances are those which “invite large individual differences in reactions that are relatively easily available, detected, and ‘correctly’ utilized by targets and their acquaintances,” suggesting the key to writing better items to is to provide “*contexts* in which trait expressions vary consistently and widely” (p. 632, emphasis added). This seems to align with what we have found here. At a glance, the most informative items (Table 1A) contain a clear contextual referent such as an object or event (reading a map, attending a concert), activity (liking philosophy or art), habitual behavior (cleaning), global self-assessment (feeling worthless), or salient situational feature (public speaking). Conversely, the least informative items largely fail to “invite large individual differences,” asking about traits that most people would likely converge on (e.g., having sympathy for the less fortunate or thinking that some aspects of their personality are likable). While De Vries et al. focused primarily on technical aspects of items, perhaps the more relevant information relates to how trait information itself is presented. One way to study this further is to collect ratings for various context-relevant criteria such as salience, importance, and observability (cf., Condon et al., 2021).

Resolving this should be a top priority for researchers to continue refining our ability to write and select high-quality items. That said, some traits may genuinely vary less in the population. If variance were one of the “causes” of higher empirical trait properties, what can we say about the traits that *generally* vary little but may have theoretically relevant atypical/divergent values? Items that assess especially maladaptive traits, for example, clearly touch upon something that many individual differences researchers would be interested in, but they may not tell us much about why people differ at the population level and what the consequences of these differences are. If so, researchers may need to consider more seriously a wider variety of different assessment techniques if they wish to more effectively assess within a normal population (Hallquist & Wright, 2014; Wright & Hallquist, 2014). Such methods may include behavioral studies that examine changing physiological markers (e.g., Dufner et al., 2015) or experience sampling studies to examine traits as distributions of states (e.g., Fleeson, 2001; Sened et al., 2018) on a day-to-day basis. Alternatively, these traits could also be studied in populations that do exhibit considerable variability on those tendencies, such as psychiatric patients or convicted criminals.

Improved study of personality assessment at high levels of specificity will lead to better questionnaires, which not only increase *academic* understanding of how personality is structured but also help applied researchers and practitioners to provide more meaningful feedback to individuals. Personality research is, fundamentally, about the accurate and meaningful depiction of our many unique traits, and if these truly are more complex than we are accustomed to thinking, then it is not for personality researchers to hide this with broad oversimplifications, but rather to find the best way to capture and describe the reality.

Future personality questionnaires aiming to more comprehensively cover the high-dimensional personality trait space will provide a win-win solution: Not only will more traits be measured for those researchers who want to consider nuances’ associations patterns, but the higher-order traits such as the Big Five and HEXACO domains will also be measured more comprehensively and with more systematically scrutinized content. Among other things, this will allow explicitly addressing the jingle-jangle problems (broad traits overlap only to the extent that their nuances overlap; Condon et al., 2021) and examining in detail where the HEXACO and Big Five domains overlap and where they diverge (cf., Thielmann et al., 2021).

### Adjustments for Cross-Time and Cross-Method Unreliability

By dividing the properties of items by their retest reliability on the one hand, and cross-rater agreement on the other, we demonstrated two different ways of partialing out random measurement error and method effects, respectively. These are not perfect techniques, requiring numerous assumptions (e.g., method variance being constant from one test moment to another across an interval, different types of informants providing the same quality of information about the target), but they provide a simple and tractable way of approximating the true values of items’ different empirical properties. Given this, a typical single item’s variance may be as heritable and consensually valid as that of the facets or even domains they were intended to measure. Even more strikingly, they appear to demonstrate near-perfect stability—as evidenced by both methods of dis-attenuation^3^—over 2 years, in which individuals can experience a great number of personal changes. What is more, these conclusions largely apply to not just items as they are, but also their unique variance, after the variance of higher order traits for which the items were written in the first place has been removed. Just a decade ago, we might have considered such a claim far-fetched, if not absurd, given the general lack of attention paid to individual personality items and the unique traits, nuances, that they capture.

### Findings Emerge Across Numerous Diverse Cultures and Languages

The unique trait properties and patterns of associations with demographic variables that items and their residuals demonstrate tend to replicate across a variety of cultural contexts, indicating that these properties are not culturally idiosyncratic. As a working hypothesis, we thus propose that items—much like the broader facets and domains they measure—may index something partly universal about human nature that transcends the unique sociocultural influences of any given country. But because most analyses here were from Western, largely wealthy nations, more diverse cross-cultural research is required. Yet the consistency of the findings with those for the NEO-PI-*R* (Mõttus et al., 2019)—which did examine a more geographically diverse set of countries, including Japan—offers at least some support to the working hypothesis.

### Interpreting Items’ Residual Variance

A key assumption of the present work is that items’ residual variance indexes information in items that is independent of higher-order variance—in other words, that it is free of *any* true score variance related to the trait it purportedly assesses. But true scores are elusive and some have argued that the residuals may in fact still contain a substantial amount of true trait variance (e.g., Allik et al., 2024). For example, a scale with four to eight items does not provide a comprehensive assessment of a broad construct, so residualizing the scale’s items for its aggregate scores leaves some true variance in the items intact. Here, however, residuals were estimated by taking into account *all* higher order variance based on 25 (24 in the HEXACO-60) facet scores, hence much more of the true trait variance was likely accounted for. This concern also does not address the evidence for nuances that does not involve any residualization: that item-based models usually (but not always) out-predict domain and facet-based models for outcomes we care about (Seeboth & Mõttus, 2018; Stewart et al., 2021). More importantly, we reiterate that the goal of this manuscript is not to denigrate existing hierarchical models of personality, but to encourage readers to consider the likely reality that meaningful personality information can be found—and leveraged—at higher levels of specificity than have been typically used.

### Limitations

While samples for cross-rater agreement and heritability estimates were quite large, we were only able to access one dataset with test–retest data that could be considered to estimate “long-term stability.” Even then, the 2-year interval in the German dataset is still a fraction of the length used to assess rank-order stability of the NEO-PI-R—where we may expect much more genuine change in 15 versus 2 years. Luckily, the SPeADy project now has another wave of data ready for analysis, meaning further estimates of stability—of the same participants—can be made soon.

We were also unable to conduct a meta-analysis for stability, as we could only locate a single sample. We also only managed to meta-analyze properties for one half of the full, 200-item HEXACO-PI-R, and could only estimate cross-rater agreement for the full version using one dataset. While in practice, the full HEXACO-200 is used much less frequently than the two shorter versions, this study technically leaves half of the items in the HEXACO-PI-R essentially un-investigated.

Finally, we note that twin modeling is just one way of approximating the differentiating role of genes in a behavioral outcome, and this is not without limitations. For example, estimates of heritability and common environment influence, being based on differences between correlations, will have somewhat more error than will the estimates of self/other agreement or long-term stability. See Verweij et al. (2012) for a review of the limitations of twin modeling.

## Conclusion

This study provides yet further support for the idea that items index a unique, specific level of the personality hierarchy below facets, with a jarring consistency among the emerging findings that is nothing short of remarkable. This work should serve as a further reminder that there is far more to personality than a few broad trait domains and prompt research on how to best maximize the breadth and precision of capturing individual differences.

## Appendix

**Table A1.** Empirical Properties for All HEXACO-100 Items

*Item*	Id	*r*_ca_		*h* ^2^		*c* ^2^		*r*_ro_		*SD*		*r*_tt_		*Inf*
		raw	res	raw	res	raw	res	raw	res	raw	res	raw	res	
If I had the opportunity, I would like to attend a classical music concert	Oaesa4	.48	.30	.50	.33	.14	.04	.78	.69	1.08	.84	.83	.66	2.00
I find it boring to discuss philosophy	Ounco8	.37	.18	.41	.24	.00	.02	.72	.54	.98	.76	.84	.71	1.39
I would be quite bored by a visit to an art gallery	Oaesa1	.44	.19	.47	.30	.00	.01	.69	.53	.99	.70	.80	.56	1.19
I clean my office or home quite frequently	Corga2	.45	.25	.40	.30	.00	.00			.94	.71	.75	.62	1.17
I enjoy looking at maps of different places	Oinqu3	.36	.22	.38	.28	.00	.00			.96	.78	.70	.61	1.05
I would like to be seen driving around in a very expensive car	Hgree5	.38	.17	.45	.26	.00	.00			.97	.68	.74	.47	.96
I tend to feel quite self-conscious when speaking in front of a group of people	Xsocb8	.37	.17	.45	.22	.00	.00			.98	.71	.72	.65	.93
I would enjoy creating a work of art, such as a novel, a song, or a painting	Ocrea6	.48	.19	.41	.16	.03	.04	.70	.52	1.07	.72	.73	.49	.93
I would be very bored by a book about the history of science and technology	Oinqu6	.29	.15	.41	.25	.00	.00			1.02	.81	.65	.52	.91
People often call me a perfectionist	Cperf4	.42	.21	.31	.21	.00	.00	.75	.54	.93	.70	.76	.62	.91
I’m interested in learning about the history and politics of other countries	Oinqu1	.42	.26	.48	.27	.01	.00	.58	.43	.95	.74	.66	.52	.87
I wouldn’t spend my time reading a book of poetry	Oaesa3	.30	.14	.43	.31	.00	.00			1.03	.82	.60	.49	.86
If I knew that I could never get caught, I would be willing to steal a million dollars	Hfair1	.40	.13	.35	.22	.19	.19	.69	.44	1.04	.73	.79	.48	.80
People often joke with me about the messiness of my room or desk	Corga6	.48	.19	.23	.13	.00	.07			1.05	.74	.73	.54	.70
I feel like crying when I see other people crying	Esent1	.39	.14	.35	.23	.00	.01	.62	.46	.92	.70	.75	.51	.63
In social situations, I’m usually the one who makes the first move	Xsocb3	.43	.18	.39	.30	.00	.09	.59	.37	.86	.59	.76	.48	.58
I’ve never really enjoyed looking through an encyclopedia	Oinqu8	.28	.11	.39	.23	.03	.06	.64	.54	.92	.72	.64	.46	.57
I don’t mind doing jobs that involve dangerous work	Efear4	.37	.12	.42	.25	.00	.01			.91	.67	.69	.47	.55
People sometimes tell me that I’m too stubborn	Aflex1	.31	.18	.27	.21	.00	.00	.62	.51	.91	.74	.68	.57	.55
I rarely, if ever, have trouble sleeping due to stress or anxiety	Eanxi6	.28	.16	.30	.20		.01			.97	.81	.64	.54	.55
Sometimes I like to just watch the wind as it blows through the trees	Oaesa7	.29	.15	.44	.21	.00	.05			.88	.73	.61	.51	.55
I plan ahead and organize things, to avoid scrambling at the last minute	Corga3	.41	.20	.28	.24	.00	.01	.67	.53	.92	.70	.60	.36	.52
People have often told me that I have a good imagination	Ocrea7	.30	.15	.36	.26	.00	.00	.62	.54	.76	.61	.71	.53	.49
I try to give generously to those in need	Alt4	.28	.19	.50	.40	.00	.00			.70	.59	.60	.42	.46
I would feel afraid if I had to travel in bad weather conditions	Efear1	.34	.17	.36	.22	.07	.16	.55	.45	.92	.75	.64	.52	.45
I prefer jobs that involve active social interaction to those that involve working alone	Xsoci5	.33	.14	.31	.12	.02	.12	.66	.53	.83	.65	.75	.55	.43
I would get a lot of pleasure from owning expensive luxury goods	Hgree7	.38	.11	.35	.21	.00	.01	.67	.51	.94	.65	.68	.38	.41
I feel that I am an unpopular person	Xsses5	.33	.15	.39	.24	.07	.08	.66	.50	.77	.56	.71	.50	.41
I sometimes feel that I am a worthless person	Xsses8	.33	.12	.36	.17	.01	.02	.61	.39	.93	.64	.77	.52	.40
I think of myself as a somewhat eccentric person	Ounco6	.20	.11	.38	.28	.00	.00			.81	.70	.66	.57	.39
I am energetic nearly all the time	Xlive2	.38	.18	.40	.17	.00	.00			.84	.60	.69	.42	.34
I would like to live in a very expensive, high-class neighborhood	Hgree4	.35	.11	.40	.17	.00	.00			.90	.62	.72	.45	.31
When I’m in a group of people, I’m often the one who speaks on behalf of the group	Xsocb4	.42	.13	.39	.11	.00	.11	.69	.47	.87	.58	.68	.41	.30
I would like a job that requires following a routine rather than being creative	Ocrea2	.28	.13	.30	.18	.00	.00			.85	.68	.70	.52	.23
I don’t think of myself as the artistic or creative type	Ocrea8	.37	.12	.36	.16	.00	.15	.41	.33	.98	.70	.74	.40	.17
I avoid making “small talk” with people	Xsoci2	.21	.09	.35	.17	.00	.00			.83	.67	.71	.56	.17
I do only the minimum amount of work needed to get by	Cdili6	.33	.15	.35	.19	.03	.04	.53	.42	.80	.63	.70	.43	.17
I’d be tempted to use counterfeit money, if I were sure I could get away with it	Hfair8	.35	.07	.29	.18	.09	.13	.63	.35	.95	.64	.73	.40	.13
When it comes to physical danger, I am very fearful	Efear7	.35	.10	.29	.17	.00	.08	.65	.49	.87	.66	.60	.39	.13
The first thing that I always do in a new place is to make friends	Xsoci6	.36	.15	.30	.16	.00	.02	.62	.48	.79	.59	.65	.41	.13
People often tell me that I should try to cheer up	Xlive4	.35	.14	.35	.17	.00	.00			.80	.57	.66	.50	.12
I feel strong emotions when someone close to me is going away for a long time	Esent3	.29	.12	.39	.23	.11	.14	.51	.39	.78	.62	.62	.50	.08
When working, I sometimes have difficulties due to being disorganized	Corga8	.35	.10	.27	.16	.00	.00	.62	.44	.87	.62	.64	.44	.08
I would be tempted to buy stolen property if I were financially tight	Hfair4	.28	.07	.34	.16	.09	.04			.90	.67	.65	.46	.08
I rarely express my opinions in group meetings	Xsocb2	.36	.11	.34	.14	.02	.13	.61	.38	.89	.64	.57	.39	.04
Even in an emergency I wouldn’t feel like panicking	Efear8	.29	.09	.30	.14	.00	.00	.57	.43	.84	.65	.69	.43	.03
People think of me as someone who has a quick temper	Apati2	.35	.11	.22	.13	.09	.18	.57	.45	.81	.58	.74	.49	.02
I enjoy having lots of people around to talk with	Xsoci3	.33	.11	.34	.23	.09	.00			.78	.56	.66	.40	.00
People sometimes tell me that I am too critical of others	Agent4	.29	.11	.27	.14	.00	.00	.55	.39	.85	.66	.66	.47	−.02
I sometimes can’t help worrying about little things	Eanxi1	.31	.09	.32	.15	.08	.08	.62	.34	.88	.65	.62	.35	−.04
I rarely discuss my problems with other people	Edepe8	.32	.10	.28	.05	.17	.16			.89	.65	.69	.47	−.04
I remain unemotional even in situations where most people get very sentimental	Esent7	.37	.10	.30	.14	.00	.07	.56	.31	.82	.59	.68	.47	−.06
I often check my work over repeatedly to find any mistakes	Cperf1	.33	.12	.27	.16	.00	.01			.82	.62	.60	.45	−.07
I rarely feel anger, even when people treat me quite badly	Apati3	.26	.07	.32	.16	.00	.00			.86	.63	.63	.43	−.10
Having a lot of money is not especially important to me	Hgree2	.29	.10	.27	.26	.00	.08	.47	.31	.80	.63	.64	.48	−.14
When I suffer from a painful experience, I need someone to make me feel comfortable	Edepe3	.33	.07	.28	.16	.00	.02	.59	.36	.85	.60	.64	.35	−.14
I find it hard to keep my temper when people insult me	Apati6	.24	.06	.26	.24	.00	.00			.85	.64	.64	.35	−.19
People see me as a hard-hearted person	Alt8	.18	.07	.30	.17	.00	.00			.77	.63	.64	.56	−.20
On most days, I feel cheerful and optimistic	Xlive3	.36	.09	.37	.15	.00	.05	.57	.31	.72	.47	.74	.33	−.22
I often push myself very hard when trying to achieve a goal	Cdili2	.30	.10	.33	.21	.00	.04	.59	.47	.72	.54	.59	.25	−.23
I feel reasonably satisfied with myself overall	Xsses1	.33	.14	.34	.14	.06	.04	.48	.27	.69	.50	.75	.49	−.23
My attitude toward people who have treated me badly is “forgive and forget”	Aforg3	.27	.09	.27	.15	.00	.01	.56	.42	.80	.59	.66	.35	−.24
I like people who have unconventional views	Ounco5	.23	.09	.34	.20	.03	.01	.57	.44	.63	.52	.58	.51	−.25
I want people to know that I am an important person of high status	Hmode8	.19	.06	.25	.11	.00	.04	.58	.44	.75	.57	.74	.47	−.26
I prefer to do whatever comes to mind, rather than stick to a plan	Cprud8	.32	.13	.26	.20	.07	.08	.51	.32	.79	.62	.58	.37	−.26
Most people are more upbeat and dynamic than I generally am	Xlive7	.31	.09	.31	.16	.09	.10	.55	.38	.78	.54	.63	.37	−.26
I worry a lot less than most people do	Eanxi4	.35	.08	.25	.07	.00	.02	.60	.32	.86	.61	.65	.38	−.27
When working, I often set ambitious goals for myself	Cdili1	.27	.09	.33	.04	.00	.07			.72	.54	.69	.50	−.32
I am an ordinary person who is no better than others	Hmode2	.20	.07	.28	.12	.14	.09			.79	.63	.62	.44	−.35
If I want something from someone, I will laugh at that person’’s worst jokes	Hsinc5	.17	.08	.23	.12	.05	.02	.52	.36	.75	.62	.70	.40	−.42
When someone I know well is unhappy, I can almost feel that persons pain myself	Esent2	.20	.04	.29	.11	.00	.06			.70	.54	.70	.49	−.42
I make decisions based on the feeling of the moment rather than on careful thought	Cprud2	.25	.06	.23	.13	.00	.01	.56	.42	.81	.62	.58	.24	−.44
Often when I set a goal, I end up quitting without having reached it	Cdili5	.22	.06	.28	.06	.00	.00			.74	.56	.69	.49	−.45
I think that I am entitled to more respect than the average person is	Hmode6	.18	.07	.26	.18	.09	.16	.52	.40	.70	.56	.63	.38	−.47
Whenever I feel worried about something, I want to share my concern with another person	Edepe7	.30	.04	.30	.12	.00	.00			.80	.53	.57	.30	−.51
It wouldn’t bother me to harm someone I didn’t like	Alt7	.26	.09	.25	.13	.00	.05			.77	.60	.56	.33	−.53
I rarely hold a grudge, even against people who have badly wronged me	Aforg1	.25	.08	.30	.13	.00	.05	.47	.23	.86	.64	.55	.25	−.53
If someone has cheated me once, I will always feel suspicious of that person	Aforg7	.17	.07	.24	.15	.00	.00			.73	.61	.58	.42	−.54
I always try to be accurate in my work, even at the expense of time	Cperf3	.30	.09	.20	.13	.00	.00	.58	.38	.74	.53	.56	.31	−.60
I can handle difficult situations without needing emotional support from anyone else	Edepe6	.28	.08	.23	.10	.00	.01	.52	.26	.83	.60	.53	.30	−.62
I make a lot of mistakes because I don’t think before I act	Cprud3	.25	.08	.24	.09	.03	.12	.54	.34	.74	.56	.58	.30	−.63
If I want something from a person I dislike, I will act very nicely toward that person in order to get it	Hsinc1	.13	.06	.21	.14	.00	.00			.80	.68	.57	.29	−.64
When people tell me that I’m wrong, my first reaction is to argue with them	Aflex7	.22	.09	.17	.15	.02	.03	.45	.37	.78	.64	.52	.29	−.64
I would never accept a bribe, even if it were very large	Hfair6	.16	.02	.17	.08	.17	.06	.38	.25	.86	.71	.70	.45	−.64
Even when people make a lot of mistakes, I rarely say anything negative	Agent7	.21	.07	.22	.14	.02	.00	.43	.25	.75	.60	.59	.40	−.65
Most people tend to get angry more quickly than I do	Apati4	.29	.07	.20	.09	.00	.00	.51	.31	.77	.56	.59	.31	−.65
I find it hard to compromise with people when I really think I’m right	Aflex8	.18	.07	.17	.15	.00	.00			.79	.65	.53	.31	−.65
I find it hard to fully forgive someone who has done something mean to me	Aforg8	.21	.04	.28	.06	.04	.15			.82	.59	.61	.25	−.67
I get very anxious when waiting to hear about an important decision	Eanxi8	.23	.10	.25	.17	.00	.00			.70	.56	.54	.33	−.68
I am usually quite flexible in my opinions when people disagree with me	Aflex5	.18	.07	.19	.08	.07	.17	.47	.38	.74	.62	.55	.37	−.68
When working on something, I don’t pay much attention to small details	Cperf2	.24	.08	.23	.13	.00	.02	.49	.29	.73	.58	.55	.34	−.69
I think that paying attention to radical ideas is a waste of time	Ounco2	.16	.07	.24	.14	.07	.03	.42	.34	.69	.62	.54	.47	−.75
I tend to be lenient in judging other people	Agent6	.21	.07	.22	.12	.00	.00	.49	.31	.72	.57	.54	.41	−.79
I have sympathy for people who are less fortunate than I am	Alt3	.19	.04	.25	.11	.06	.03			.61	.47	.64	.42	−.81
I wouldn’t use flattery to get a raise or promotion at work, even if I thought it would succeed	Hsinc4	.14	.03	.25	.18	.00	.00	.42	.27	.84	.71	.48	.20	−.81
I generally accept peoples faults without complaining about them	Agent5	.18	.06	.30	.15	.00	.00			.72	.57	.48	.26	−.83
I think that most people like some aspects of my personality	Xsses3	.14	.04	.26	.19	.00				.50	.42	.56	.42	−1.05
I wouldn’t pretend to like someone just to get that person to do favors for me	Hsinc6	.10	.04	.20	.11	.08	.08	.42	.26	.81	.69	.39	.15	−1.06
I don’t allow my impulses to govern my behavior	Cprud4	.19	.04	.23	.11	.00	.02			.75	.59	.47	.25	−1.06
I wouldn’t want people to treat me as though I were superior to them	Hmode3	.16	.04	.19	.01	.19	.12			.70	.57	.46	.25	−1.29

*Note.* Items are ordered by decreasing magnitude of overall informativeness. Estimates for items’ residual variance are italicized. *r*_ca_ = cross-rater agreement. *r*_ro_ = rank-order stability. *h*^2^ = heritability. *c*^2^ = shared environmental influence. *SD* = standard deviation. *r*_tt_ = test–retest reliability.

## Notes

1. Given that median imputation can introduce bias (e.g., if the missingness is not completely random), we also analyzed stability, and self-/informant-, and demographic data using only complete cases (i.e., listwise deletion). The results were nearly identical, so we report the larger median-imputed samples here; results of the alternative analysis can be found in the Online Supplement.

2. This is true to the extent that the assumption that method effects are equal across items holds; see McCrae (2015) for a more detailed discussion of this topic.

3. We note that *r*_tt_-adjusted values for any given item should be interpreted with caution for a few reasons. First, they are adjusted using out-of-sample *r*_tt_ estimates, which are always noisier and thus may track worse with validity criteria. Second, there is as-yet no clear guidance on which retest interval best approximates retest reliability, and *r*_tt_ estimates decline almost linearly up to a month. If, say, 1-week estimates are more accurate indicators of “true” *r*_tt_, then our estimates would be *underestimates*, meaning adjustments to empirical properties would unduly inflate them. We thus aimed to approximate the *average* level of empirical properties when adjusting for measurement error.
